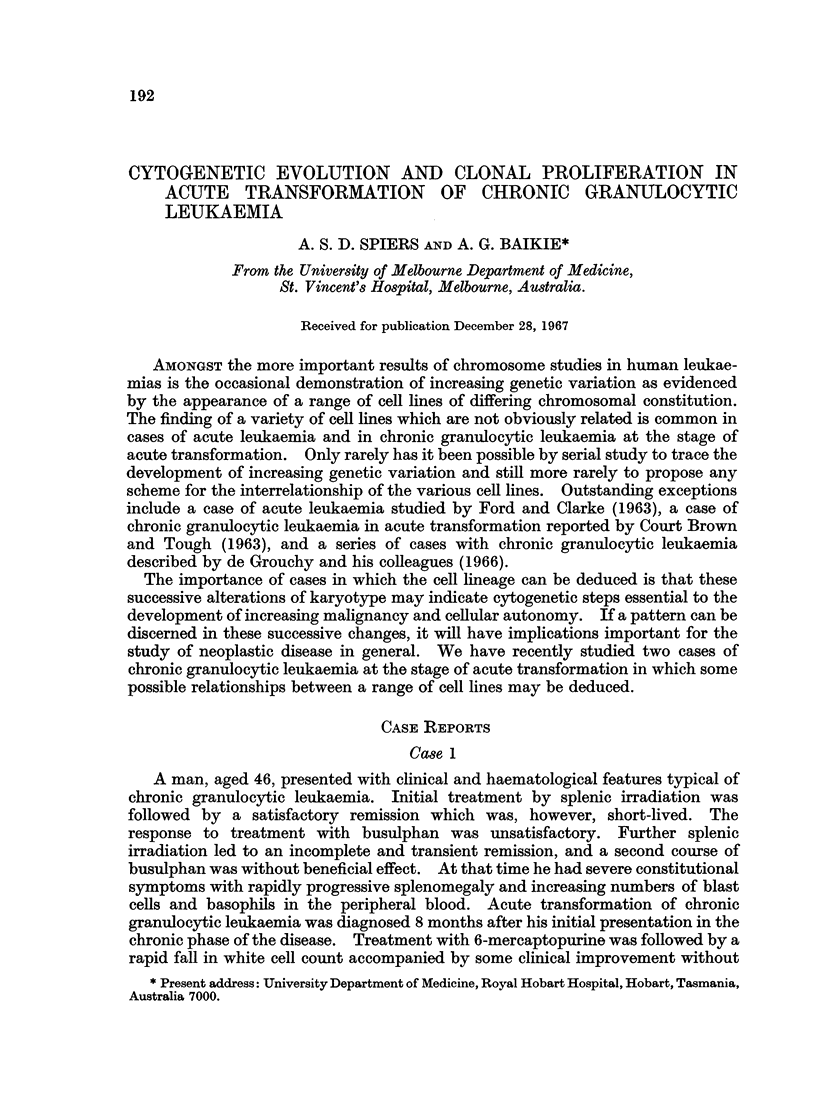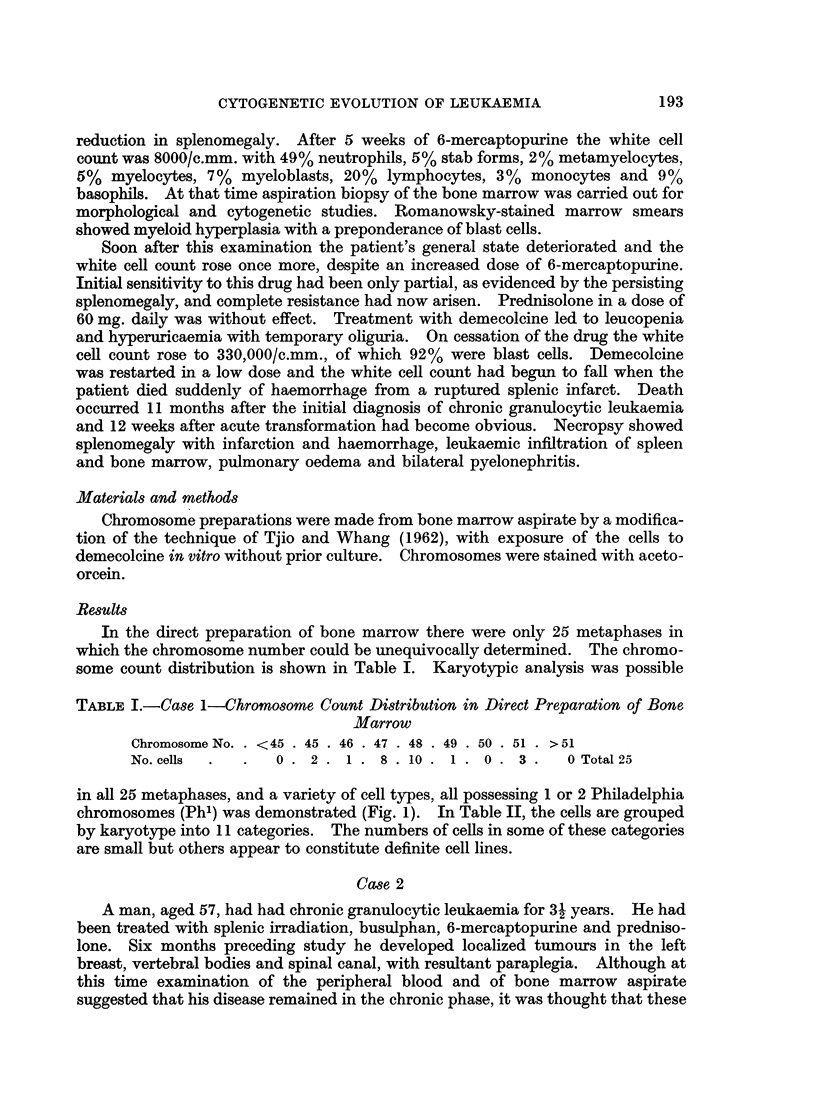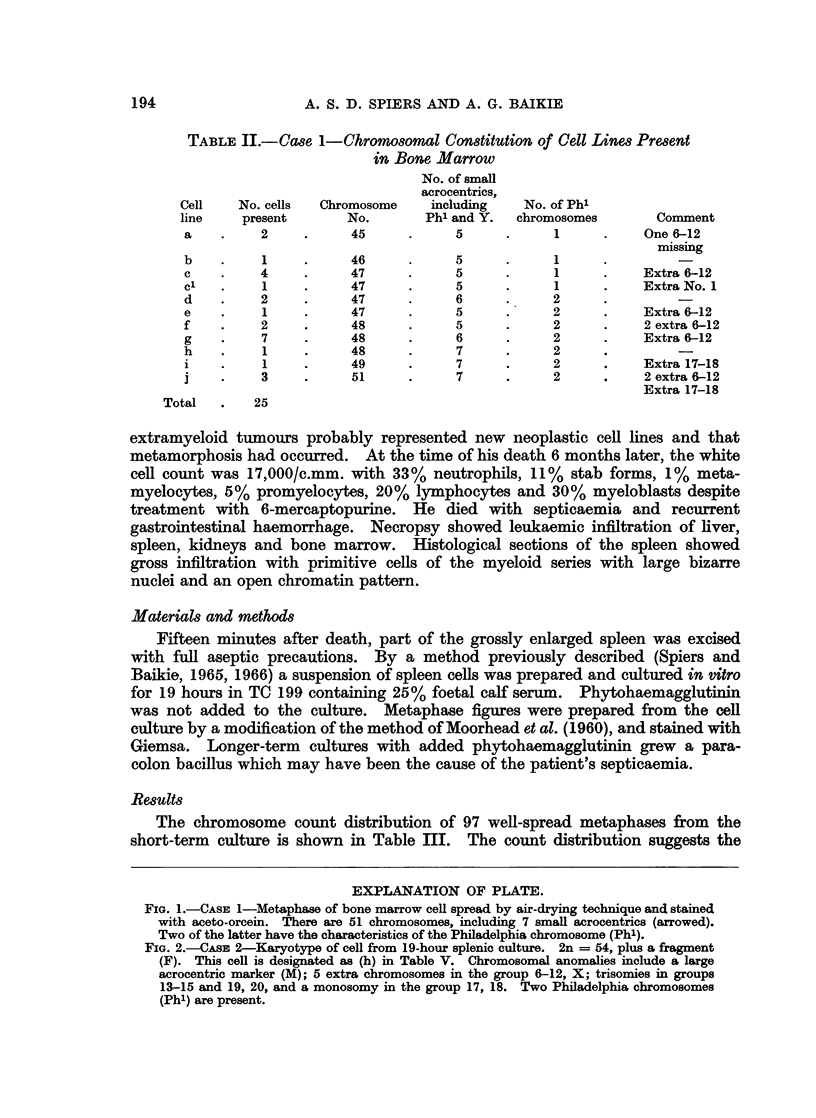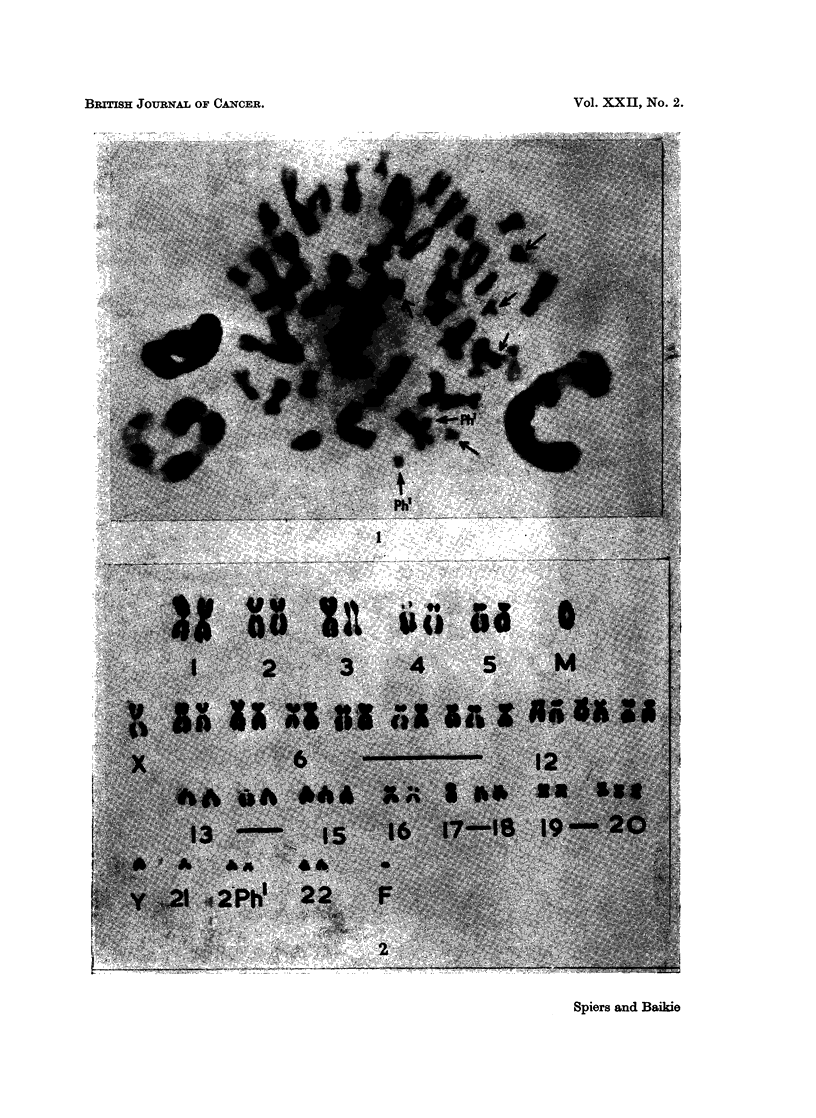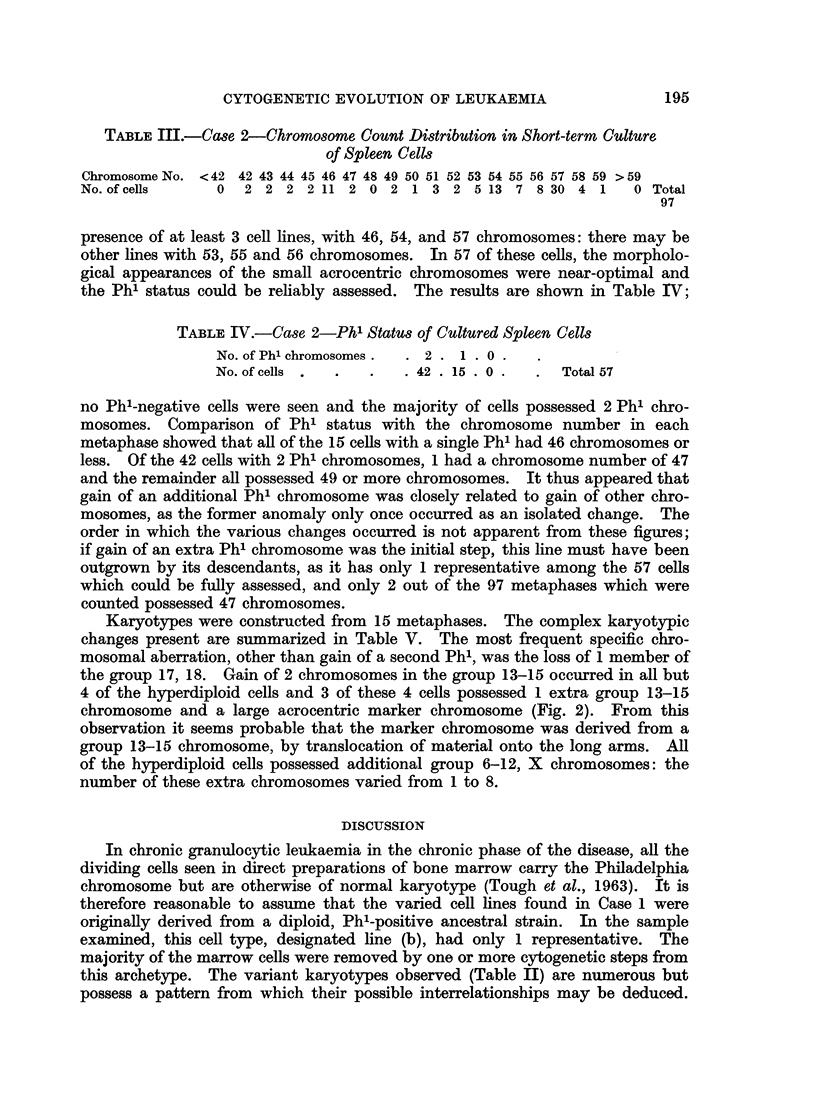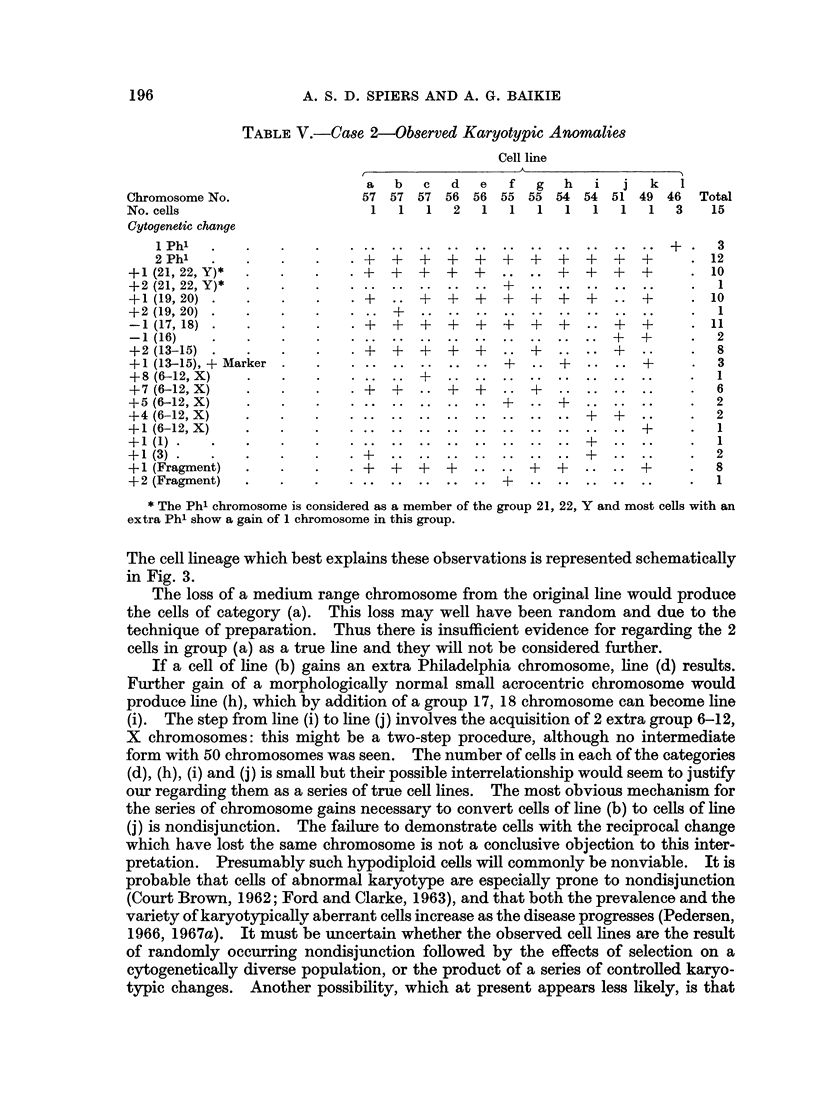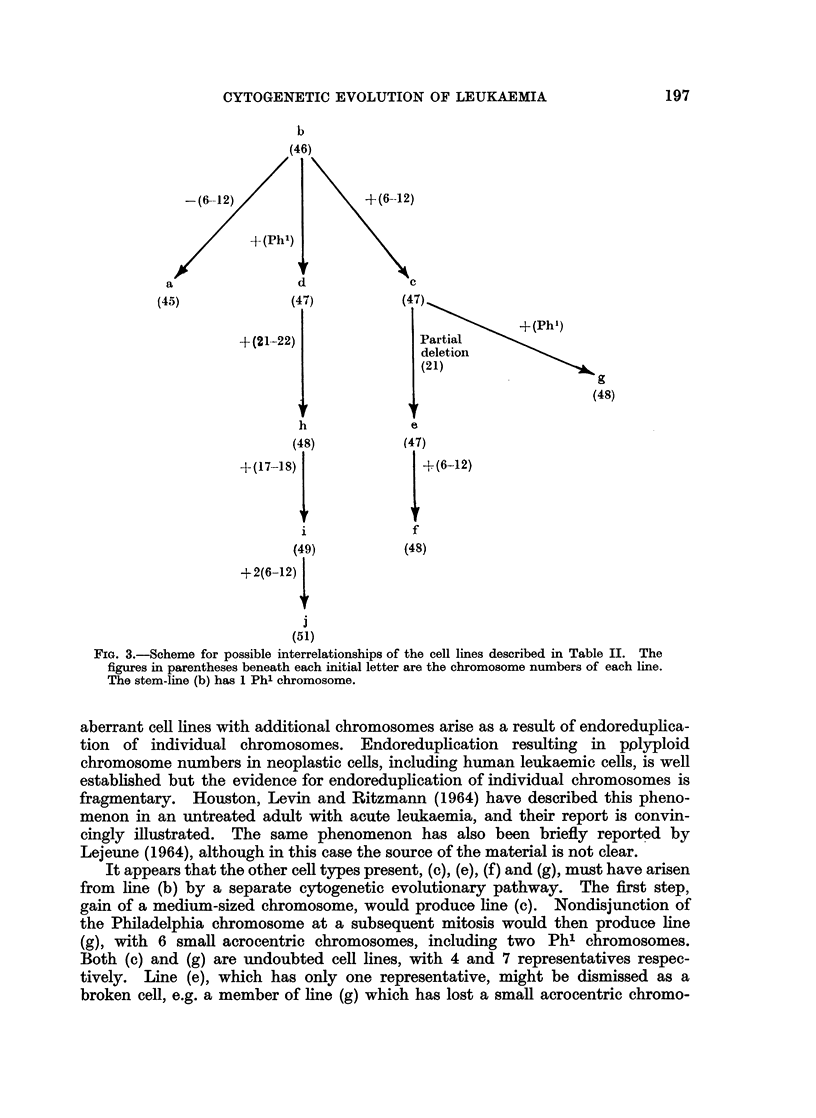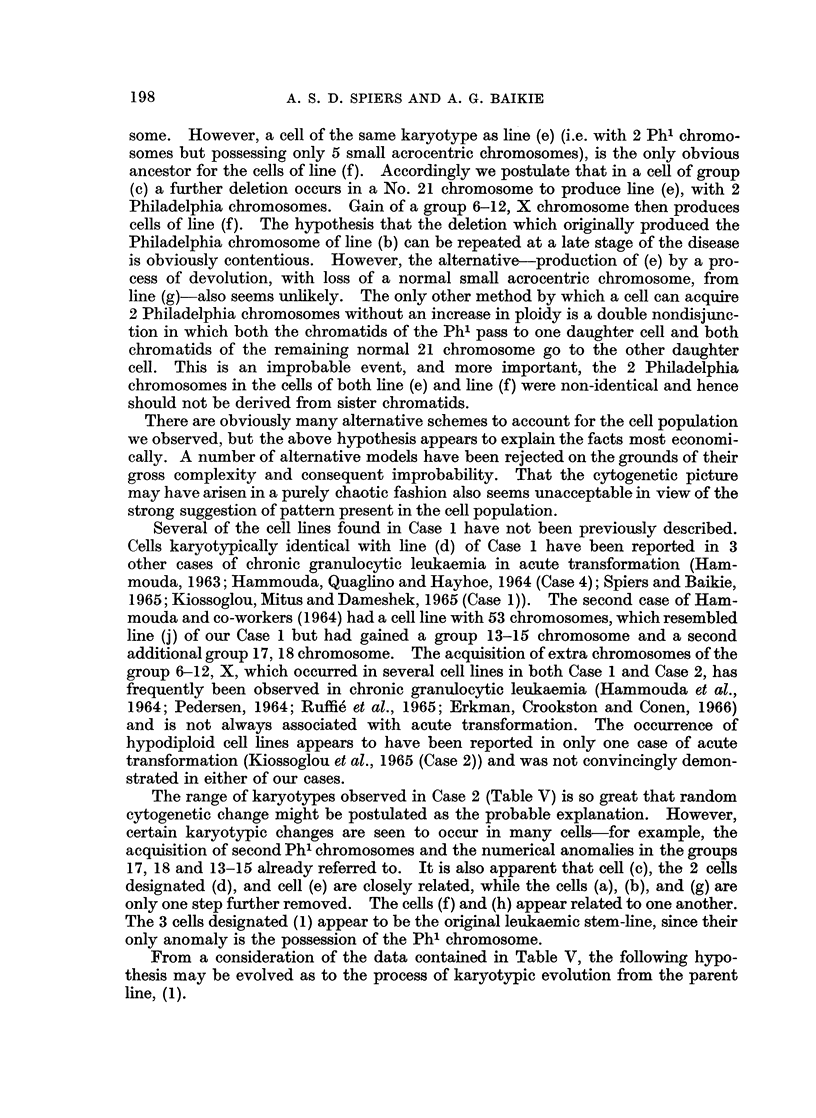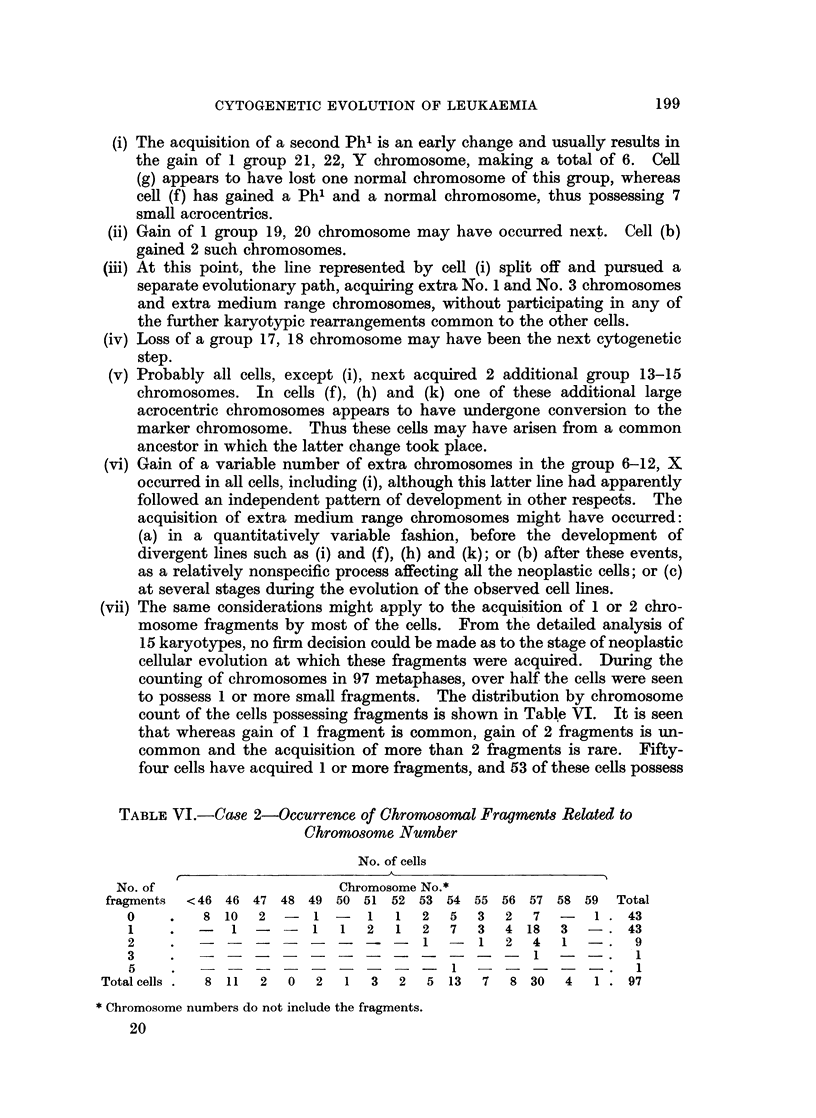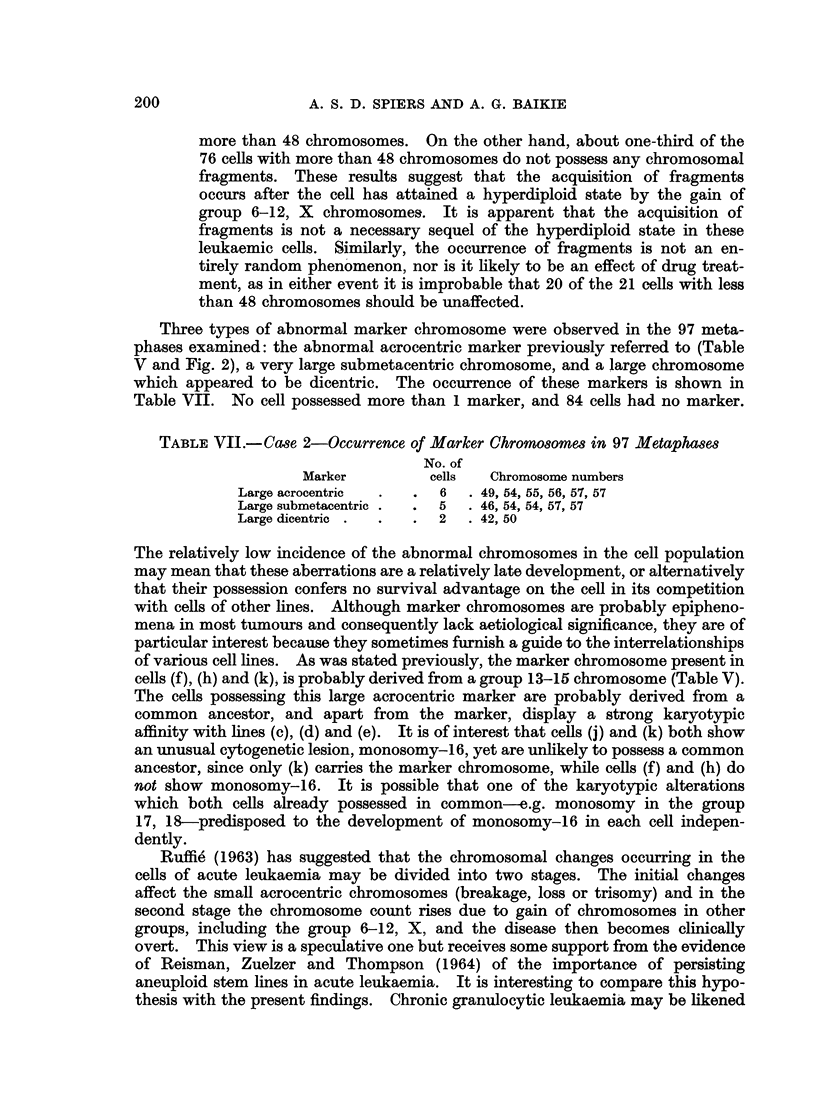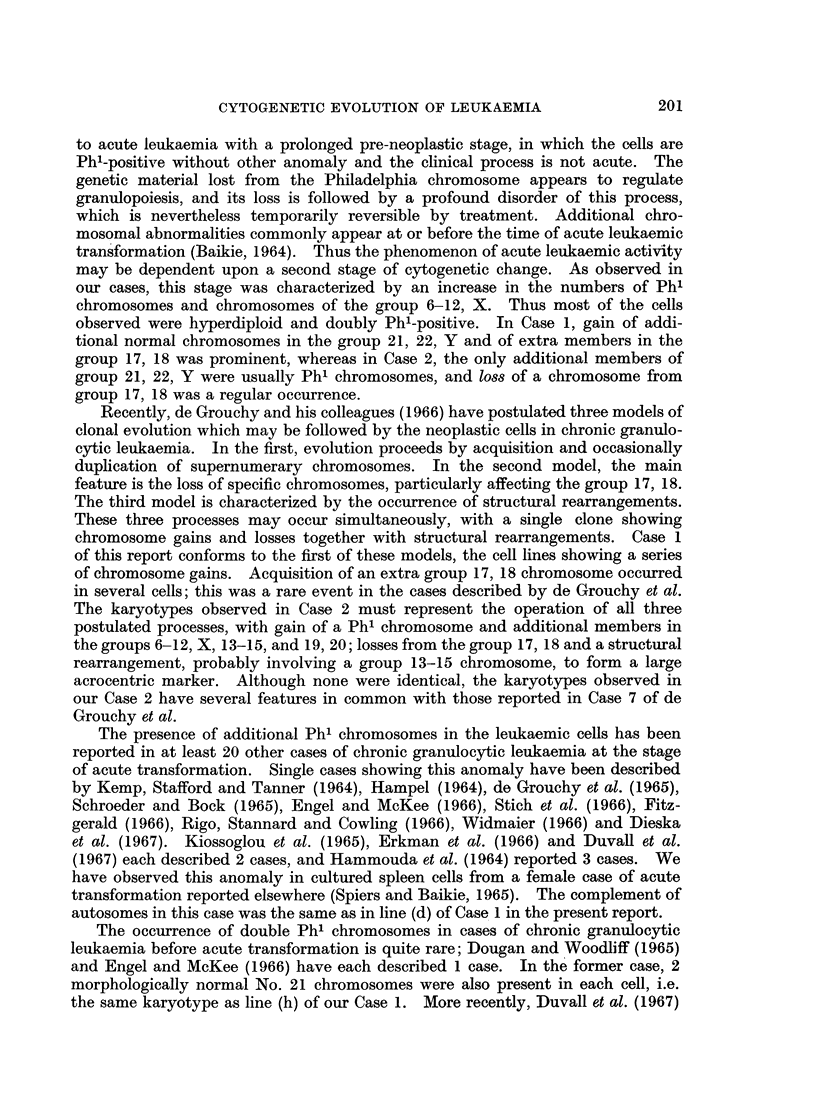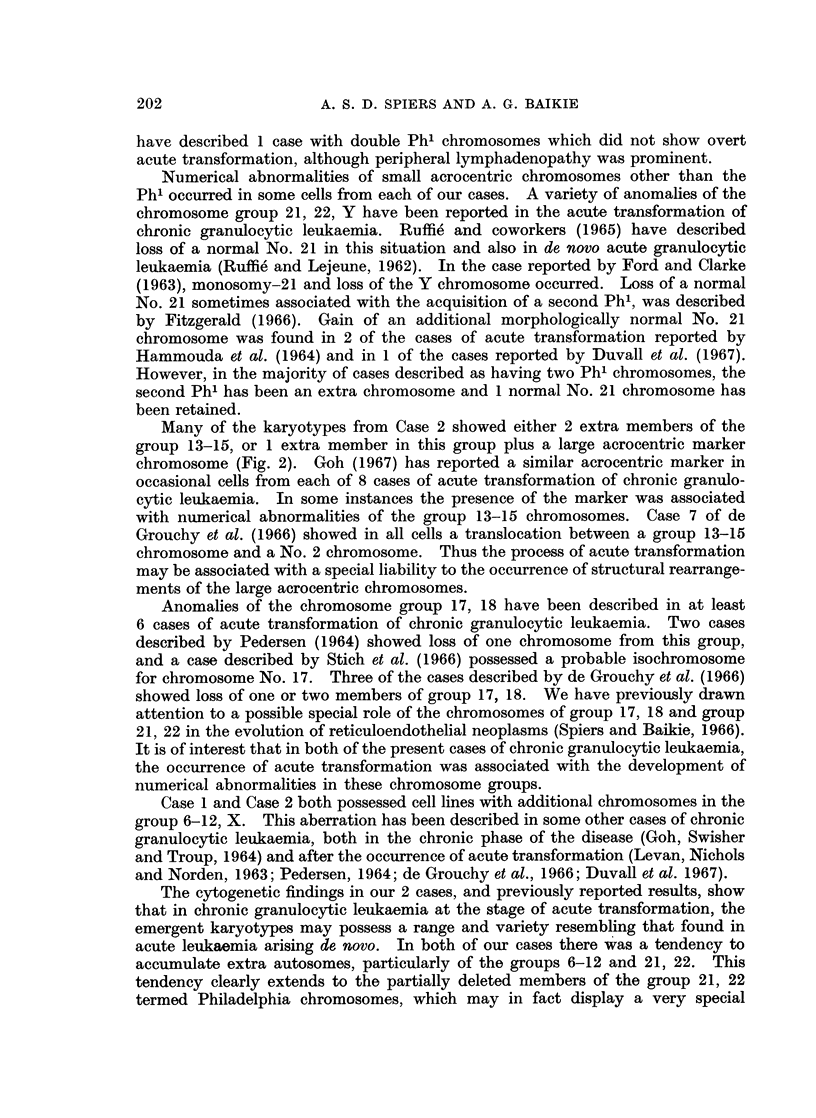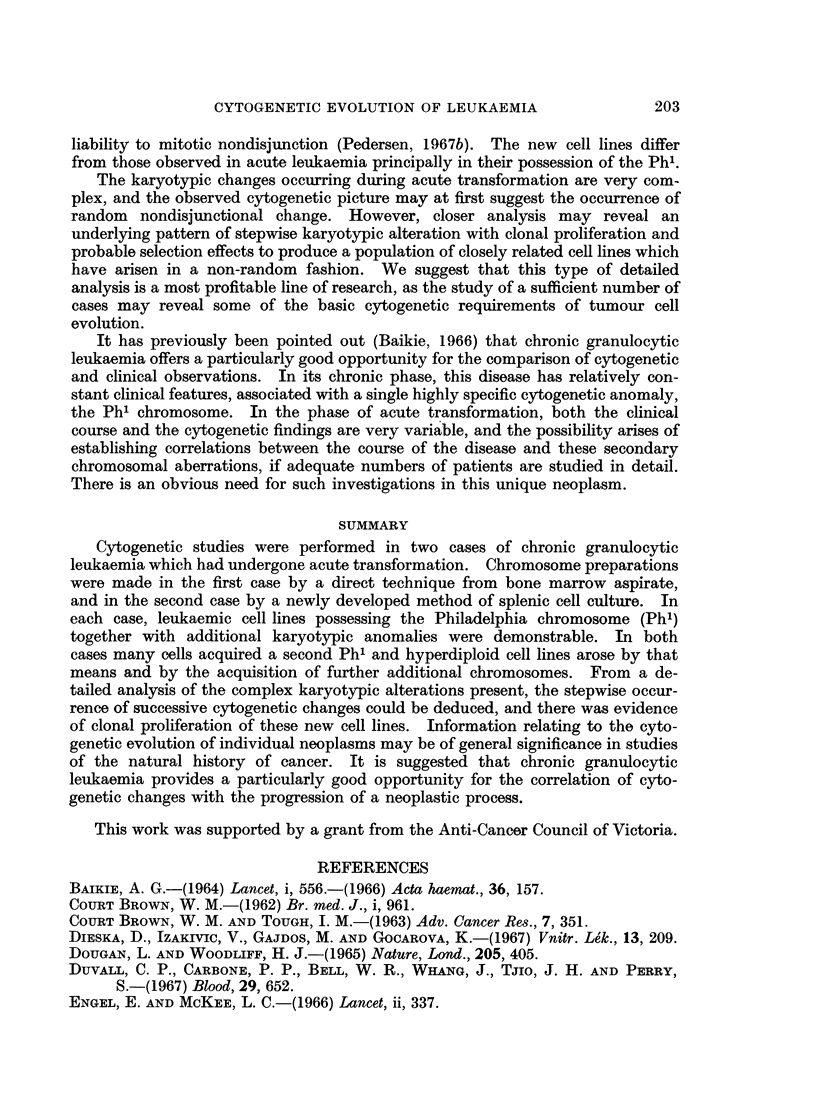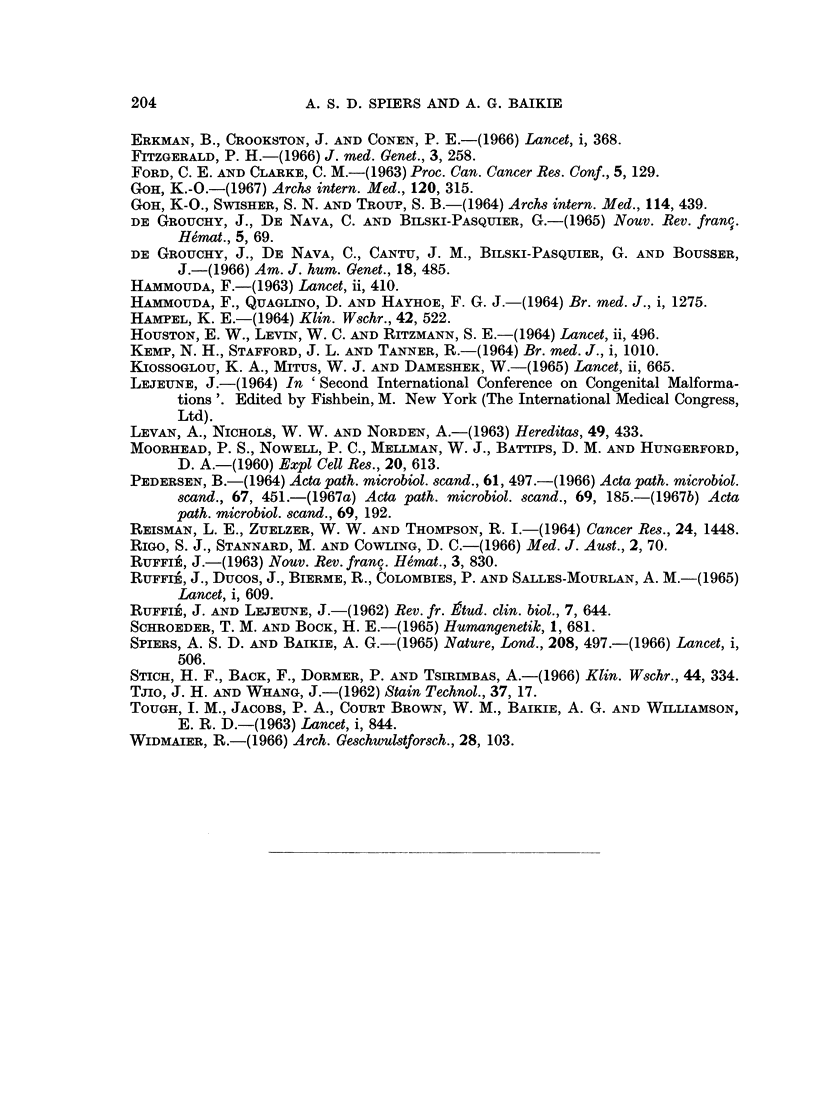# Cytogenetic evolution and clonal proliferation in acute transformation of chronic granulocytic leukaemia.

**DOI:** 10.1038/bjc.1968.27

**Published:** 1968-06

**Authors:** A. S. Spiers, A. G. Baikie

## Abstract

**Images:**


### CYTOGENETIC EVOLUTION AND CLONAL PROLIFERATION IN ACUTE TRANSFORMATION OF CHRONIC GRANULOCYTIC LEUKAEMIA

A. S. D. SPIERS AND A. G. BAIKIE*

*From the University of Melbourne Department of Medicine, St. Vincent's Hospital, Melbourne, Australia.*

Received for publication December 28, 1967

AMONGST the more important results of chromosome studies in human leukaemias is the occasional demonstration of increasing genetic variation as evidenced by the appearance of a range of cell lines of differing chromosomal constitution. The finding of a variety of cell lines which are not obviously related is common in cases of acute leukaemia and in chronic granulocytic leukaemia at the stage of acute transformation. Only rarely has it been possible by serial study to trace the development of increasing genetic variation and still more rarely to propose any scheme for the interrelationship of the various cell lines. Outstanding exceptions include a case of acute leukaemia studied by Ford and Clarke (1963), a case of chronic granulocytic leukaemia in acute transformation reported by Court Brown and Tough (1963), and a series of cases with chronic granulocytic leukaemia described by de Grouchy and his colleagues (1966).

The importance of cases in which the cell lineage can be deduced is that these successive alterations of karyotype may indicate cytogenetic steps essential to the development of increasing malignancy and cellular autonomy. If a pattern can be discerned in these successive changes, it will have implications important for the study of neoplastic disease in general. We have recently studied two cases of chronic granulocytic leukaemia at the stage of acute transformation in which some possible relationships between a range of cell lines may be deduced.

### CASE REPORTS

### *Case* 1

A man, aged 46, presented with clinical and haematological features typical of chronic granulocytic leukaemia. Initial treatment by splenic irradiation was followed by a satisfactory remission which was, however, short-lived. The response to treatment with busulphan was unsatisfactory. Further splenic irradiation led to an incomplete and transient remission, and a second course of busulphan was without beneficial effect. At that time he had severe constitutional symptoms with rapidly progressive splenomegaly and increasing numbers of blast cells and basophils in the peripheral blood. Acute transformation of chronic granulocytic leukaemia was diagnosed 8 months after his initial presentation in the chronic phase of the disease. Treatment with 6-mercaptopurine was followed by a rapid fall in white cell count accompanied by some clinical improvement without

* Present address: University Department of Medicine, Royal Hobart Hospital, Hobart, Tasmania, Australia 7000.

reduction in splenomegaly. After 5 weeks of 6-mercaptopurine the white cell count was 8000/c.mm. with 49% neutrophils, 5% stab forms, 2% metamyelocytes, 5% myelocytes, 7% myeloblasts, 20% lymphocytes, 3% monocytes and 9% basophils. At that time aspiration biopsy of the bone marrow was carried out for morphological and cytogenetic studies. Romanowsky-stained marrow smears showed myeloid hyperplasia with a preponderance of blast cells.

Soon after this examination the patient's general state deteriorated and the white cell count rose once more, despite an increased dose of 6-mercaptopurine. Initial sensitivity to this drug had been only partial, as evidenced by the persisting splenomegaly, and complete resistance had now arisen. Prednisolone in a dose of 60 mg. daily was without effect. Treatment with demecolcine led to leucopenia and hyperuricaemia with temporary oliguria. On cessation of the drug the white cell count rose to 330,000/c.mm., of which 92% were blast cells. Demecolcine was restarted in a low dose and the white cell count had begun to fall when the patient died suddenly of haemorrhage from a ruptured splenic infarct. Death occurred 11 months after the initial diagnosis of chronic granulocytic leukaemia and 12 weeks after acute transformation had become obvious. Necropsy showed splenomegaly with infarction and haemorrhage, leukaemic infiltration of spleen and bone marrow, pulmonary oedema and bilateral pyelonephritis.

*Materials and methods*

Chromosome preparations were made from bone marrow aspirate by a modification of the technique of Tjio and Whang (1962), with exposure of the cells to demecolcine *in vitro* without prior culture. Chromosomes were stained with aceto-orcein.

*Results*

In the direct preparation of bone marrow there were only 25 metaphases in which the chromosome number could be unequivocally determined. The chromosome count distribution is shown in Table I. Karyotypic analysis was possible

TABLE I.—*Case 1—Chromosome Count Distribution in Direct Preparation of Bone Marrow*

| Chromosome No. | <45 | 45 | 46 | 47 | 48 | 49 | 50 | 51 | >51 | |
|---|---|---|---|---|---|---|---|---|---|---|
| No. cells | 0 | 2 | 1 | 8 | 10 | 1 | 0 | 3 | 0 | Total 25 |

in all 25 metaphases, and a variety of cell types, all possessing 1 or 2 Philadelphia chromosomes ($Ph^1$) was demonstrated (Fig. 1). In Table II, the cells are grouped by karyotype into 11 categories. The numbers of cells in some of these categories are small but others appear to constitute definite cell lines.

*Case 2*

A man, aged 57, had had chronic granulocytic leukaemia for 3½ years. He had been treated with splenic irradiation, busulphan, 6-mercaptopurine and prednisolone. Six months preceding study he developed localized tumours in the left breast, vertebral bodies and spinal canal, with resultant paraplegia. Although at this time examination of the peripheral blood and of bone marrow aspirate suggested that his disease remained in the chronic phase, it was thought that these

TABLE II.—*Case 1—Chromosomal Constitution of Cell Lines Present in Bone Marrow*

| Cell line | No. cells present | Chromosome No. | No. of small acrocentrics, including $Ph^1$ and Y. | No. of $Ph^1$ chromosomes | Comment |
|---|---|---|---|---|---|
| a | 2 | 45 | 5 | 1 | One 6–12 missing |
| b | 1 | 46 | 5 | 1 | — |
| c | 4 | 47 | 5 | 1 | Extra 6–12 |
| $c^1$ | 1 | 47 | 5 | 1 | Extra No. 1 |
| d | 2 | 47 | 6 | 2 | — |
| e | 1 | 47 | 5 | 2 | Extra 6–12 |
| f | 2 | 48 | 5 | 2 | 2 extra 6–12 |
| g | 7 | 48 | 6 | 2 | Extra 6–12 |
| h | 1 | 48 | 7 | 2 | — |
| i | 1 | 49 | 7 | 2 | Extra 17–18 |
| j | 3 | 51 | 7 | 2 | 2 extra 6–12 Extra 17–18 |
| Total | 25 | | | | |

extramyeloid tumours probably represented new neoplastic cell lines and that metamorphosis had occurred. At the time of his death 6 months later, the white cell count was 17,000/c.mm. with 33% neutrophils, 11% stab forms, 1% metamyelocytes, 5% promyelocytes, 20% lymphocytes and 30% myeloblasts despite treatment with 6-mercaptopurine. He died with septicaemia and recurrent gastrointestinal haemorrhage. Necropsy showed leukaemic infiltration of liver, spleen, kidneys and bone marrow. Histological sections of the spleen showed gross infiltration with primitive cells of the myeloid series with large bizarre nuclei and an open chromatin pattern.

### *Materials and methods*

Fifteen minutes after death, part of the grossly enlarged spleen was excised with full aseptic precautions. By a method previously described (Spiers and Baikie, 1965, 1966) a suspension of spleen cells was prepared and cultured *in vitro* for 19 hours in TC 199 containing 25% foetal calf serum. Phytohaemagglutinin was not added to the culture. Metaphase figures were prepared from the cell culture by a modification of the method of Moorhead *et al.* (1960), and stained with Giemsa. Longer-term cultures with added phytohaemagglutinin grew a paracolon bacillus which may have been the cause of the patient's septicaemia.

### *Results*

The chromosome count distribution of 97 well-spread metaphases from the short-term culture is shown in Table III. The count distribution suggests the

---

EXPLANATION OF PLATE.

FIG. 1.—CASE 1—Metaphase of bone marrow cell spread by air-drying technique and stained with aceto-orcein. There are 51 chromosomes, including 7 small acrocentrics (arrowed). Two of the latter have the characteristics of the Philadelphia chromosome ($Ph^1$).

FIG. 2.—CASE 2—Karyotype of cell from 19-hour splenic culture. 2n = 54, plus a fragment (F). This cell is designated as (h) in Table V. Chromosomal anomalies include a large acrocentric marker (M); 5 extra chromosomes in the group 6–12, X; trisomies in groups 13–15 and 19, 20, and a monosomy in the group 17, 18. Two Philadelphia chromosomes ($Ph^1$) are present.

1

2

TABLE III.—*Case 2—Chromosome Count Distribution in Short-term Culture of Spleen Cells*

| Chromosome No. | <42 | 42 | 43 | 44 | 45 | 46 | 47 | 48 | 49 | 50 | 51 | 52 | 53 | 54 | 55 | 56 | 57 | 58 | 59 | >59 | |
|---|---|---|---|---|---|---|---|---|---|---|---|---|---|---|---|---|---|---|---|---|---|
| No. of cells | 0 | 2 | 2 | 2 | 2 | 11 | 2 | 0 | 2 | 1 | 3 | 2 | 5 | 13 | 7 | 8 | 30 | 4 | 1 | 0 | Total 97 |

presence of at least 3 cell lines, with 46, 54, and 57 chromosomes: there may be other lines with 53, 55 and 56 chromosomes. In 57 of these cells, the morphological appearances of the small acrocentric chromosomes were near-optimal and the $Ph^1$ status could be reliably assessed. The results are shown in Table IV;

TABLE IV.—*Case 2—$Ph^1$ Status of Cultured Spleen Cells*

| | | | | |
|---|---|---|---|---|
| No. of $Ph^1$ chromosomes | 2 | 1 | 0 | |
| No. of cells | 42 | 15 | 0 | Total 57 |

no $Ph^1$-negative cells were seen and the majority of cells possessed 2 $Ph^1$ chromosomes. Comparison of $Ph^1$ status with the chromosome number in each metaphase showed that all of the 15 cells with a single $Ph^1$ had 46 chromosomes or less. Of the 42 cells with 2 $Ph^1$ chromosomes, 1 had a chromosome number of 47 and the remainder all possessed 49 or more chromosomes. It thus appeared that gain of an additional $Ph^1$ chromosome was closely related to gain of other chromosomes, as the former anomaly only once occurred as an isolated change. The order in which the various changes occurred is not apparent from these figures; if gain of an extra $Ph^1$ chromosome was the initial step, this line must have been outgrown by its descendants, as it has only 1 representative among the 57 cells which could be fully assessed, and only 2 out of the 97 metaphases which were counted possessed 47 chromosomes.

Karyotypes were constructed from 15 metaphases. The complex karyotypic changes present are summarized in Table V. The most frequent specific chromosomal aberration, other than gain of a second $Ph^1$, was the loss of 1 member of the group 17, 18. Gain of 2 chromosomes in the group 13–15 occurred in all but 4 of the hyperdiploid cells and 3 of these 4 cells possessed 1 extra group 13–15 chromosome and a large acrocentric marker chromosome (Fig. 2). From this observation it seems probable that the marker chromosome was derived from a group 13–15 chromosome, by translocation of material onto the long arms. All of the hyperdiploid cells possessed additional group 6–12, X chromosomes: the number of these extra chromosomes varied from 1 to 8.

### DISCUSSION

In chronic granulocytic leukaemia in the chronic phase of the disease, all the dividing cells seen in direct preparations of bone marrow carry the Philadelphia chromosome but are otherwise of normal karyotype (Tough *et al.*, 1963). It is therefore reasonable to assume that the varied cell lines found in Case 1 were originally derived from a diploid, $Ph^1$-positive ancestral strain. In the sample examined, this cell type, designated line (b), had only 1 representative. The majority of the marrow cells were removed by one or more cytogenetic steps from this archetype. The variant karyotypes observed (Table II) are numerous but possess a pattern from which their possible interrelationships may be deduced.

TABLE V.—*Case 2—Observed Karyotypic Anomalies*

| | Cell line | | | | | | | | | | | | |
|---|---|---|---|---|---|---|---|---|---|---|---|---|---|
| | a | b | c | d | e | f | g | h | i | j | k | l | |
| Chromosome No. | 57 | 57 | 57 | 56 | 56 | 55 | 55 | 54 | 54 | 51 | 49 | 46 | Total |
| No. cells | 1 | 1 | 1 | 2 | 1 | 1 | 1 | 1 | 1 | 1 | 1 | 3 | 15 |
| *Cytogenetic change* | | | | | | | | | | | | | |
| 1 Ph¹ | .. | .. | .. | .. | .. | .. | .. | .. | .. | .. | .. | + | 3 |
| 2 Ph¹ | + | + | + | + | + | + | + | + | + | + | + | | 12 |
| +1 (21, 22, Y)* | + | + | + | + | + | .. | .. | + | + | + | + | | 10 |
| +2 (21, 22, Y)* | .. | .. | .. | .. | .. | + | .. | .. | .. | .. | .. | | 1 |
| +1 (19, 20) | + | .. | + | + | + | + | + | + | + | .. | + | | 10 |
| +2 (19, 20) | .. | + | .. | .. | .. | .. | .. | .. | .. | .. | .. | | 1 |
| −1 (17, 18) | + | + | + | + | + | + | + | + | .. | + | + | | 11 |
| −1 (16) | .. | .. | .. | .. | .. | .. | .. | .. | .. | + | + | | 2 |
| +2 (13–15) | + | + | + | + | + | .. | + | .. | .. | + | .. | | 8 |
| +1 (13–15), + Marker | .. | .. | .. | .. | .. | + | .. | + | .. | .. | + | | 3 |
| +8 (6–12, X) | .. | .. | + | .. | .. | .. | .. | .. | .. | .. | .. | | 1 |
| +7 (6–12, X) | + | + | .. | + | + | .. | + | .. | .. | .. | .. | | 6 |
| +5 (6–12, X) | .. | .. | .. | .. | .. | + | .. | + | .. | .. | .. | | 2 |
| +4 (6–12, X) | .. | .. | .. | .. | .. | .. | .. | .. | + | + | .. | | 2 |
| +1 (6–12, X) | .. | .. | .. | .. | .. | .. | .. | .. | .. | .. | + | | 1 |
| +1 (1) | .. | .. | .. | .. | .. | .. | .. | .. | + | .. | .. | | 1 |
| +1 (3) | + | .. | .. | .. | .. | .. | .. | .. | + | .. | .. | | 2 |
| +1 (Fragment) | + | + | + | + | .. | .. | + | + | .. | .. | + | | 8 |
| +2 (Fragment) | .. | .. | .. | .. | .. | + | .. | .. | .. | .. | .. | | 1 |

* The Ph¹ chromosome is considered as a member of the group 21, 22, Y and most cells with an extra Ph¹ show a gain of 1 chromosome in this group.

The cell lineage which best explains these observations is represented schematically in Fig. 3.

The loss of a medium range chromosome from the original line would produce the cells of category (a). This loss may well have been random and due to the technique of preparation. Thus there is insufficient evidence for regarding the 2 cells in group (a) as a true line and they will not be considered further.

If a cell of line (b) gains an extra Philadelphia chromosome, line (d) results. Further gain of a morphologically normal small acrocentric chromosome would produce line (h), which by addition of a group 17, 18 chromosome can become line (i). The step from line (i) to line (j) involves the acquisition of 2 extra group 6–12, X chromosomes: this might be a two-step procedure, although no intermediate form with 50 chromosomes was seen. The number of cells in each of the categories (d), (h), (i) and (j) is small but their possible interrelationship would seem to justify our regarding them as a series of true cell lines. The most obvious mechanism for the series of chromosome gains necessary to convert cells of line (b) to cells of line (j) is nondisjunction. The failure to demonstrate cells with the reciprocal change which have lost the same chromosome is not a conclusive objection to this interpretation. Presumably such hypodiploid cells will commonly be nonviable. It is probable that cells of abnormal karyotype are especially prone to nondisjunction (Court Brown, 1962; Ford and Clarke, 1963), and that both the prevalence and the variety of karyotypically aberrant cells increase as the disease progresses (Pedersen, 1966, 1967*a*). It must be uncertain whether the observed cell lines are the result of randomly occurring nondisjunction followed by the effects of selection on a cytogenetically diverse population, or the product of a series of controlled karyotypic changes. Another possibility, which at present appears less likely, is that

FIG. 3.—Scheme for possible interrelationships of the cell lines described in Table II. The figures in parentheses beneath each initial letter are the chromosome numbers of each line. The stem-line (b) has 1 $Ph^1$ chromosome.

aberrant cell lines with additional chromosomes arise as a result of endoreduplication of individual chromosomes. Endoreduplication resulting in polyploid chromosome numbers in neoplastic cells, including human leukaemic cells, is well established but the evidence for endoreduplication of individual chromosomes is fragmentary. Houston, Levin and Ritzmann (1964) have described this phenomenon in an untreated adult with acute leukaemia, and their report is convincingly illustrated. The same phenomenon has also been briefly reported by Lejeune (1964), although in this case the source of the material is not clear.

It appears that the other cell types present, (c), (e), (f) and (g), must have arisen from line (b) by a separate cytogenetic evolutionary pathway. The first step, gain of a medium-sized chromosome, would produce line (c). Nondisjunction of the Philadelphia chromosome at a subsequent mitosis would then produce line (g), with 6 small acrocentric chromosomes, including two $Ph^1$ chromosomes. Both (c) and (g) are undoubted cell lines, with 4 and 7 representatives respectively. Line (e), which has only one representative, might be dismissed as a broken cell, e.g. a member of line (g) which has lost a small acrocentric chromosome. However, a cell of the same karyotype as line (e) (i.e. with 2 Ph[1] chromosomes but possessing only 5 small acrocentric chromosomes), is the only obvious ancestor for the cells of line (f). Accordingly we postulate that in a cell of group (c) a further deletion occurs in a No. 21 chromosome to produce line (e), with 2 Philadelphia chromosomes. Gain of a group 6–12, X chromosome then produces cells of line (f). The hypothesis that the deletion which originally produced the Philadelphia chromosome of line (b) can be repeated at a late stage of the disease is obviously contentious. However, the alternative—production of (e) by a process of devolution, with loss of a normal small acrocentric chromosome, from line (g)—also seems unlikely. The only other method by which a cell can acquire 2 Philadelphia chromosomes without an increase in ploidy is a double nondisjunction in which both the chromatids of the Ph[1] pass to one daughter cell and both chromatids of the remaining normal 21 chromosome go to the other daughter cell. This is an improbable event, and more important, the 2 Philadelphia chromosomes in the cells of both line (e) and line (f) were non-identical and hence should not be derived from sister chromatids.

There are obviously many alternative schemes to account for the cell population we observed, but the above hypothesis appears to explain the facts most economically. A number of alternative models have been rejected on the grounds of their gross complexity and consequent improbability. That the cytogenetic picture may have arisen in a purely chaotic fashion also seems unacceptable in view of the strong suggestion of pattern present in the cell population.

Several of the cell lines found in Case 1 have not been previously described. Cells karyotypically identical with line (d) of Case 1 have been reported in 3 other cases of chronic granulocytic leukaemia in acute transformation (Hammouda, 1963; Hammouda, Quaglino and Hayhoe, 1964 (Case 4); Spiers and Baikie, 1965; Kiossoglou, Mitus and Dameshek, 1965 (Case 1)). The second case of Hammouda and co-workers (1964) had a cell line with 53 chromosomes, which resembled line (j) of our Case 1 but had gained a group 13–15 chromosome and a second additional group 17, 18 chromosome. The acquisition of extra chromosomes of the group 6–12, X, which occurred in several cell lines in both Case 1 and Case 2, has frequently been observed in chronic granulocytic leukaemia (Hammouda *et al.*, 1964; Pedersen, 1964; Ruffié *et al.*, 1965; Erkman, Crookston and Conen, 1966) and is not always associated with acute transformation. The occurrence of hypodiploid cell lines appears to have been reported in only one case of acute transformation (Kiossoglou *et al.*, 1965 (Case 2)) and was not convincingly demonstrated in either of our cases.

The range of karyotypes observed in Case 2 (Table V) is so great that random cytogenetic change might be postulated as the probable explanation. However, certain karyotypic changes are seen to occur in many cells—for example, the acquisition of second Ph[1] chromosomes and the numerical anomalies in the groups 17, 18 and 13–15 already referred to. It is also apparent that cell (c), the 2 cells designated (d), and cell (e) are closely related, while the cells (a), (b), and (g) are only one step further removed. The cells (f) and (h) appear related to one another. The 3 cells designated (1) appear to be the original leukaemic stem-line, since their only anomaly is the possession of the Ph[1] chromosome.

From a consideration of the data contained in Table V, the following hypothesis may be evolved as to the process of karyotypic evolution from the parent line, (1).

(i) The acquisition of a second Ph[1] is an early change and usually results in the gain of 1 group 21, 22, Y chromosome, making a total of 6. Cell (g) appears to have lost one normal chromosome of this group, whereas cell (f) has gained a Ph[1] and a normal chromosome, thus possessing 7 small acrocentrics.

(ii) Gain of 1 group 19, 20 chromosome may have occurred next. Cell (b) gained 2 such chromosomes.

(iii) At this point, the line represented by cell (i) split off and pursued a separate evolutionary path, acquiring extra No. 1 and No. 3 chromosomes and extra medium range chromosomes, without participating in any of the further karyotypic rearrangements common to the other cells.

(iv) Loss of a group 17, 18 chromosome may have been the next cytogenetic step.

(v) Probably all cells, except (i), next acquired 2 additional group 13–15 chromosomes. In cells (f), (h) and (k) one of these additional large acrocentric chromosomes appears to have undergone conversion to the marker chromosome. Thus these cells may have arisen from a common ancestor in which the latter change took place.

(vi) Gain of a variable number of extra chromosomes in the group 6–12, X occurred in all cells, including (i), although this latter line had apparently followed an independent pattern of development in other respects. The acquisition of extra medium range chromosomes might have occurred: (a) in a quantitatively variable fashion, before the development of divergent lines such as (i) and (f), (h) and (k); or (b) after these events, as a relatively nonspecific process affecting all the neoplastic cells; or (c) at several stages during the evolution of the observed cell lines.

(vii) The same considerations might apply to the acquisition of 1 or 2 chromosome fragments by most of the cells. From the detailed analysis of 15 karyotypes, no firm decision could be made as to the stage of neoplastic cellular evolution at which these fragments were acquired. During the counting of chromosomes in 97 metaphases, over half the cells were seen to possess 1 or more small fragments. The distribution by chromosome count of the cells possessing fragments is shown in Table VI. It is seen that whereas gain of 1 fragment is common, gain of 2 fragments is uncommon and the acquisition of more than 2 fragments is rare. Fifty-four cells have acquired 1 or more fragments, and 53 of these cells possess

TABLE VI.—*Case 2—Occurrence of Chromosomal Fragments Related to Chromosome Number*

| No. of fragments | No. of cells: Chromosome No.* <46 | 46 | 47 | 48 | 49 | 50 | 51 | 52 | 53 | 54 | 55 | 56 | 57 | 58 | 59 | Total |
|---|---|---|---|---|---|---|---|---|---|---|---|---|---|---|---|---|
| 0 | 8 | 10 | 2 | — | 1 | — | 1 | 1 | 2 | 5 | 3 | 2 | 7 | — | 1 | 43 |
| 1 | — | 1 | — | — | 1 | 1 | 2 | 1 | 2 | 7 | 3 | 4 | 18 | 3 | — | 43 |
| 2 | — | — | — | — | — | — | — | — | 1 | — | 1 | 2 | 4 | 1 | — | 9 |
| 3 | — | — | — | — | — | — | — | — | — | — | — | — | 1 | — | — | 1 |
| 5 | — | — | — | — | — | — | — | — | — | 1 | — | — | — | — | — | 1 |
| Total cells | 8 | 11 | 2 | 0 | 2 | 1 | 3 | 2 | 5 | 13 | 7 | 8 | 30 | 4 | 1 | 97 |

* Chromosome numbers do not include the fragments.

more than 48 chromosomes. On the other hand, about one-third of the 76 cells with more than 48 chromosomes do not possess any chromosomal fragments. These results suggest that the acquisition of fragments occurs after the cell has attained a hyperdiploid state by the gain of group 6–12, X chromosomes. It is apparent that the acquisition of fragments is not a necessary sequel of the hyperdiploid state in these leukaemic cells. Similarly, the occurrence of fragments is not an entirely random phenomenon, nor is it likely to be an effect of drug treatment, as in either event it is improbable that 20 of the 21 cells with less than 48 chromosomes should be unaffected.

Three types of abnormal marker chromosome were observed in the 97 metaphases examined: the abnormal acrocentric marker previously referred to (Table V and Fig. 2), a very large submetacentric chromosome, and a large chromosome which appeared to be dicentric. The occurrence of these markers is shown in Table VII. No cell possessed more than 1 marker, and 84 cells had no marker.

TABLE VII.—*Case 2—Occurrence of Marker Chromosomes in 97 Metaphases*

| Marker | No. of cells | Chromosome numbers |
|---|---|---|
| Large acrocentric | 6 | 49, 54, 55, 56, 57, 57 |
| Large submetacentric | 5 | 46, 54, 54, 57, 57 |
| Large dicentric | 2 | 42, 50 |

The relatively low incidence of the abnormal chromosomes in the cell population may mean that these aberrations are a relatively late development, or alternatively that their possession confers no survival advantage on the cell in its competition with cells of other lines. Although marker chromosomes are probably epiphenomena in most tumours and consequently lack aetiological significance, they are of particular interest because they sometimes furnish a guide to the interrelationships of various cell lines. As was stated previously, the marker chromosome present in cells (f), (h) and (k), is probably derived from a group 13–15 chromosome (Table V). The cells possessing this large acrocentric marker are probably derived from a common ancestor, and apart from the marker, display a strong karyotypic affinity with lines (c), (d) and (e). It is of interest that cells (j) and (k) both show an unusual cytogenetic lesion, monosomy–16, yet are unlikely to possess a common ancestor, since only (k) carries the marker chromosome, while cells (f) and (h) do *not* show monosomy–16. It is possible that one of the karyotypic alterations which both cells already possessed in common—e.g. monosomy in the group 17, 18—predisposed to the development of monosomy–16 in each cell independently.

Ruffié (1963) has suggested that the chromosomal changes occurring in the cells of acute leukaemia may be divided into two stages. The initial changes affect the small acrocentric chromosomes (breakage, loss or trisomy) and in the second stage the chromosome count rises due to gain of chromosomes in other groups, including the group 6–12, X, and the disease then becomes clinically overt. This view is a speculative one but receives some support from the evidence of Reisman, Zuelzer and Thompson (1964) of the importance of persisting aneuploid stem lines in acute leukaemia. It is interesting to compare this hypothesis with the present findings. Chronic granulocytic leukaemia may be likened to acute leukaemia with a prolonged pre-neoplastic stage, in which the cells are Ph[1]-positive without other anomaly and the clinical process is not acute. The genetic material lost from the Philadelphia chromosome appears to regulate granulopoiesis, and its loss is followed by a profound disorder of this process, which is nevertheless temporarily reversible by treatment. Additional chromosomal abnormalities commonly appear at or before the time of acute leukaemic transformation (Baikie, 1964). Thus the phenomenon of acute leukaemic activity may be dependent upon a second stage of cytogenetic change. As observed in our cases, this stage was characterized by an increase in the numbers of Ph[1] chromosomes and chromosomes of the group 6–12, X. Thus most of the cells observed were hyperdiploid and doubly Ph[1]-positive. In Case 1, gain of additional normal chromosomes in the group 21, 22, Y and of extra members in the group 17, 18 was prominent, whereas in Case 2, the only additional members of group 21, 22, Y were usually Ph[1] chromosomes, and *loss* of a chromosome from group 17, 18 was a regular occurrence.

Recently, de Grouchy and his colleagues (1966) have postulated three models of clonal evolution which may be followed by the neoplastic cells in chronic granulocytic leukaemia. In the first, evolution proceeds by acquisition and occasionally duplication of supernumerary chromosomes. In the second model, the main feature is the loss of specific chromosomes, particularly affecting the group 17, 18. The third model is characterized by the occurrence of structural rearrangements. These three processes may occur simultaneously, with a single clone showing chromosome gains and losses together with structural rearrangements. Case 1 of this report conforms to the first of these models, the cell lines showing a series of chromosome gains. Acquisition of an extra group 17, 18 chromosome occurred in several cells; this was a rare event in the cases described by de Grouchy *et al.* The karyotypes observed in Case 2 must represent the operation of all three postulated processes, with gain of a Ph[1] chromosome and additional members in the groups 6–12, X, 13–15, and 19, 20; losses from the group 17, 18 and a structural rearrangement, probably involving a group 13–15 chromosome, to form a large acrocentric marker. Although none were identical, the karyotypes observed in our Case 2 have several features in common with those reported in Case 7 of de Grouchy *et al.*

The presence of additional Ph[1] chromosomes in the leukaemic cells has been reported in at least 20 other cases of chronic granulocytic leukaemia at the stage of acute transformation. Single cases showing this anomaly have been described by Kemp, Stafford and Tanner (1964), Hampel (1964), de Grouchy *et al.* (1965), Schroeder and Bock (1965), Engel and McKee (1966), Stich *et al.* (1966), Fitzgerald (1966), Rigo, Stannard and Cowling (1966), Widmaier (1966) and Dieska *et al.* (1967). Kiossoglou *et al.* (1965), Erkman *et al.* (1966) and Duvall *et al.* (1967) each described 2 cases, and Hammouda *et al.* (1964) reported 3 cases. We have observed this anomaly in cultured spleen cells from a female case of acute transformation reported elsewhere (Spiers and Baikie, 1965). The complement of autosomes in this case was the same as in line (d) of Case 1 in the present report.

The occurrence of double Ph[1] chromosomes in cases of chronic granulocytic leukaemia before acute transformation is quite rare; Dougan and Woodliff (1965) and Engel and McKee (1966) have each described 1 case. In the former case, 2 morphologically normal No. 21 chromosomes were also present in each cell, i.e. the same karyotype as line (h) of our Case 1. More recently, Duvall *et al.* (1967)

have described 1 case with double Ph[1] chromosomes which did not show overt acute transformation, although peripheral lymphadenopathy was prominent.

Numerical abnormalities of small acrocentric chromosomes other than the Ph[1] occurred in some cells from each of our cases. A variety of anomalies of the chromosome group 21, 22, Y have been reported in the acute transformation of chronic granulocytic leukaemia. Ruffié and coworkers (1965) have described loss of a normal No. 21 in this situation and also in *de novo* acute granulocytic leukaemia (Ruffié and Lejeune, 1962). In the case reported by Ford and Clarke (1963), monosomy–21 and loss of the Y chromosome occurred. Loss of a normal No. 21 sometimes associated with the acquisition of a second Ph[1], was described by Fitzgerald (1966). Gain of an additional morphologically normal No. 21 chromosome was found in 2 of the cases of acute transformation reported by Hammouda *et al.* (1964) and in 1 of the cases reported by Duvall *et al.* (1967). However, in the majority of cases described as having two Ph[1] chromosomes, the second Ph[1] has been an extra chromosome and 1 normal No. 21 chromosome has been retained.

Many of the karyotypes from Case 2 showed either 2 extra members of the group 13–15, or 1 extra member in this group plus a large acrocentric marker chromosome (Fig. 2). Goh (1967) has reported a similar acrocentric marker in occasional cells from each of 8 cases of acute transformation of chronic granulocytic leukaemia. In some instances the presence of the marker was associated with numerical abnormalities of the group 13–15 chromosomes. Case 7 of de Grouchy *et al.* (1966) showed in all cells a translocation between a group 13–15 chromosome and a No. 2 chromosome. Thus the process of acute transformation may be associated with a special liability to the occurrence of structural rearrangements of the large acrocentric chromosomes.

Anomalies of the chromosome group 17, 18 have been described in at least 6 cases of acute transformation of chronic granulocytic leukaemia. Two cases described by Pedersen (1964) showed loss of one chromosome from this group, and a case described by Stich *et al.* (1966) possessed a probable isochromosome for chromosome No. 17. Three of the cases described by de Grouchy *et al.* (1966) showed loss of one or two members of group 17, 18. We have previously drawn attention to a possible special role of the chromosomes of group 17, 18 and group 21, 22 in the evolution of reticuloendothelial neoplasms (Spiers and Baikie, 1966). It is of interest that in both of the present cases of chronic granulocytic leukaemia, the occurrence of acute transformation was associated with the development of numerical abnormalities in these chromosome groups.

Case 1 and Case 2 both possessed cell lines with additional chromosomes in the group 6–12, X. This aberration has been described in some other cases of chronic granulocytic leukaemia, both in the chronic phase of the disease (Goh, Swisher and Troup, 1964) and after the occurrence of acute transformation (Levan, Nichols and Norden, 1963; Pedersen, 1964; de Grouchy *et al.*, 1966; Duvall *et al.* 1967).

The cytogenetic findings in our 2 cases, and previously reported results, show that in chronic granulocytic leukaemia at the stage of acute transformation, the emergent karyotypes may possess a range and variety resembling that found in acute leukaemia arising *de novo*. In both of our cases there was a tendency to accumulate extra autosomes, particularly of the groups 6–12 and 21, 22. This tendency clearly extends to the partially deleted members of the group 21, 22 termed Philadelphia chromosomes, which may in fact display a very special liability to mitotic nondisjunction (Pedersen, 1967*b*). The new cell lines differ from those observed in acute leukaemia principally in their possession of the Ph[1].

The karyotypic changes occurring during acute transformation are very complex, and the observed cytogenetic picture may at first suggest the occurrence of random nondisjunctional change. However, closer analysis may reveal an underlying pattern of stepwise karyotypic alteration with clonal proliferation and probable selection effects to produce a population of closely related cell lines which have arisen in a non-random fashion. We suggest that this type of detailed analysis is a most profitable line of research, as the study of a sufficient number of cases may reveal some of the basic cytogenetic requirements of tumour cell evolution.

It has previously been pointed out (Baikie, 1966) that chronic granulocytic leukaemia offers a particularly good opportunity for the comparison of cytogenetic and clinical observations. In its chronic phase, this disease has relatively constant clinical features, associated with a single highly specific cytogenetic anomaly, the Ph[1] chromosome. In the phase of acute transformation, both the clinical course and the cytogenetic findings are very variable, and the possibility arises of establishing correlations between the course of the disease and these secondary chromosomal aberrations, if adequate numbers of patients are studied in detail. There is an obvious need for such investigations in this unique neoplasm.

### SUMMARY

Cytogenetic studies were performed in two cases of chronic granulocytic leukaemia which had undergone acute transformation. Chromosome preparations were made in the first case by a direct technique from bone marrow aspirate, and in the second case by a newly developed method of splenic cell culture. In each case, leukaemic cell lines possessing the Philadelphia chromosome (Ph[1]) together with additional karyotypic anomalies were demonstrable. In both cases many cells acquired a second Ph[1] and hyperdiploid cell lines arose by that means and by the acquisition of further additional chromosomes. From a detailed analysis of the complex karyotypic alterations present, the stepwise occurrence of successive cytogenetic changes could be deduced, and there was evidence of clonal proliferation of these new cell lines. Information relating to the cytogenetic evolution of individual neoplasms may be of general significance in studies of the natural history of cancer. It is suggested that chronic granulocytic leukaemia provides a particularly good opportunity for the correlation of cytogenetic changes with the progression of a neoplastic process.

This work was supported by a grant from the Anti-Cancer Council of Victoria.

### REFERENCES

BAIKIE, A. G.—(1964) *Lancet*, i, 556.—(1966) *Acta haemat.*, **36**, 157.
COURT BROWN, W. M.—(1962) *Br. med. J.*, i, 961.
COURT BROWN, W. M. AND TOUGH, I. M.—(1963) *Adv. Cancer Res.*, **7**, 351.
DIESKA, D., IZAKIVIC, V., GAJDOS, M. AND GOCAROVA, K.—(1967) *Vnitr. Lék.*, **13**, 209.
DOUGAN, L. AND WOODLIFF, H. J.—(1965) *Nature, Lond.*, **205**, 405.
DUVALL, C. P., CARBONE, P. P., BELL, W. R., WHANG, J., TJIO, J. H. AND PERRY, S.—(1967) *Blood*, **29**, 652.
ENGEL, E. AND MCKEE, L. C.—(1966) *Lancet*, ii, 337.

Erkman, B., Crookston, J. and Conen, P. E.—(1966) *Lancet*, i, 368.
Fitzgerald, P. H.—(1966) *J. med. Genet.*, **3**, 258.
Ford, C. E. and Clarke, C. M.—(1963) *Proc. Can. Cancer Res. Conf.*, **5**, 129.
Goh, K.-O.—(1967) *Archs intern. Med.*, **120**, 315.
Goh, K-O., Swisher, S. N. and Troup, S. B.—(1964) *Archs intern. Med.*, **114**, 439.
de Grouchy, J., De Nava, C. and Bilski-Pasquier, G.—(1965) *Nouv. Rev. franç. Hémat.*, **5**, 69.
de Grouchy, J., De Nava, C., Cantu, J. M., Bilski-Pasquier, G. and Bousser, J.—(1966) *Am. J. hum. Genet.*, **18**, 485.
Hammouda, F.—(1963) *Lancet*, ii, 410.
Hammouda, F., Quaglino, D. and Hayhoe, F. G. J.—(1964) *Br. med. J.*, i, 1275.
Hampel, K. E.—(1964) *Klin. Wschr.*, **42**, 522.
Houston, E. W., Levin, W. C. and Ritzmann, S. E.—(1964) *Lancet*, ii, 496.
Kemp, N. H., Stafford, J. L. and Tanner, R.—(1964) *Br. med. J.*, i, 1010.
Kiossoglou, K. A., Mitus, W. J. and Dameshek, W.—(1965) *Lancet*, ii, 665.
Lejeune, J.—(1964) *In* ' Second International Conference on Congenital Malformations '. Edited by Fishbein, M. New York (The International Medical Congress, Ltd).
Levan, A., Nichols, W. W. and Norden, A.—(1963) *Hereditas*, **49**, 433.
Moorhead, P. S., Nowell, P. C., Mellman, W. J., Battips, D. M. and Hungerford, D. A.—(1960) *Expl Cell Res.*, **20**, 613.
Pedersen, B.—(1964) *Acta path. microbiol. scand.*, **61**, 497.—(1966) *Acta path. microbiol. scand.*, **67**, 451.—(1967*a*) *Acta path. microbiol. scand.*, **69**, 185.—(1967*b*) *Acta path. microbiol. scand.*, **69**, 192.
Reisman, L. E., Zuelzer, W. W. and Thompson, R. I.—(1964) *Cancer Res.*, **24**, 1448.
Rigo, S. J., Stannard, M. and Cowling, D. C.—(1966) *Med. J. Aust.*, **2**, 70.
Ruffié, J.—(1963) *Nouv. Rev. franç. Hémat.*, **3**, 830.
Ruffié, J., Ducos, J., Bierme, R., Colombies, P. and Salles-Mourlan, A. M.—(1965) *Lancet*, i, 609.
Ruffié, J. and Lejeune, J.—(1962) *Rev. fr. Étud. clin. biol.*, **7**, 644.
Schroeder, T. M. and Bock, H. E.—(1965) *Humangenetik*, **1**, 681.
Spiers, A. S. D. and Baikie, A. G.—(1965) *Nature, Lond.*, **208**, 497.—(1966) *Lancet*, i, 506.
Stich, H. F., Back, F., Dormer, P. and Tsirimbas, A.—(1966) *Klin. Wschr.*, **44**, 334.
Tjio, J. H. and Whang, J.—(1962) *Stain Technol.*, **37**, 17.
Tough, I. M., Jacobs, P. A., Court Brown, W. M., Baikie, A. G. and Williamson, E. R. D.—(1963) *Lancet*, i, 844.
Widmaier, R.—(1966) *Arch. Geschwulstforsch.*, **28**, 103.